# Limb-belt resisted sprint training improves physical fitness and ball-throw velocity in pubertal handball players

**DOI:** 10.5114/biolsport.2025.139855

**Published:** 2024-07-08

**Authors:** Emna Makni, Firas Zghal, Raouf Hammami, Mohamed Abdelkader, Raghad Tarwneh, Mohamed Elloumi

**Affiliations:** 1 University of Sousse, Research Laboratory of Exercise Physiology and Pathophysiology (LR19ES09), Faculty of Medicine of Sousse. Sousse, Tunisia; 2 Research Laboratory “Education, Motor Skills, Sports and Health (EM2S, LR15JS01)”, Higher Institute of Sport and Physical Education of Sfax, University of Sfax, Sfax, Tunisia; 3 Department of Statistics and Operations Research, Faculty of Sciences, King Saud University, Riyadh, Saudi Arabia; 4 Sport Sciences and Diagnostics Research Group, GSD-HPE Department, Prince Sultan University, Riyadh, Saudi Arabia

**Keywords:** Resisted training, Distributed load, Vertical load, Performance, Handball

## Abstract

The study assessed the effect of six weeks of biweekly upper and lower limbs’ weighted-belt resisted sprint training (BRST) and weighted-vest resisted sprint training (VRST), or normal sprint training (NST) on muscle strength, speed, change of direction and handball-throwing velocity in young handball players. Twenty-seven pubertal male handball players aged 14.4 years were randomly assigned into BRST (n = 9), VRST (n = 8), and NST (n = 8) groups. Sprint ability (10-m and 30-m), squat jump (SJ), countermovement jump (CMJ), free-arm countermovement jump (CMJFA), standing long jump (SLJ), Five-jump test (FJT), change of direction and handball-throw velocity were assessed before and after a 6-week training in-season program. Within-group interactions showed that BRST improved all tests’ performances (moderate-to-large). VRST improved sprint, SLJ, FJT and handball-throw velocity performances (small-to-large). NST improved only the change of direction performances (moderate). Between-groups comparison revealed that BRST improved all testing performances, except change of direction, compared with NST (large) and improved 30-m sprint, CMJFA, FJT and handball-throw velocity performance compared with VRST (moderate-to-large). In addition, VRST improved 30-m sprint, SJ and handball-throw velocity performances compared with NST (moderate-to-large). Throwing performance changes correlated with changes in sprint time and horizontal and vertical jump abilities (r = 0.40 to r = 0.69; p < 0.01). We conclude that while both resisted sprint training improved players’ sprint, jumping and handball-throw performances, substantial improvements were recorded with the BRST compared to VRST and NST. Thus, BRST could be recommended to male U15 handball players as a valuable training method for developing physical fitness and skill performances.

## INTRODUCTION

Handball is an intermittent team sport characterized by frequent body contact and the need for repeated explosive muscular contractions, including jumping, acceleration, sprinting, turning, changing pace and direction, and handball-throw velocity [1]. To achieve success at the highest level, a well-developed and comprehensive training program oriented toward the sport’s specificities is crucial. The principle of training specificity requires that exercises should aim to replicate the demands of the respective activity. Therefore, coaches and practitioners need to identify the physical and physiological demands of handball to improve performance through targeted training programs. Numerous studies have highlighted the importance of jumping, sprinting, and change of direction abilities as vital performance components in high-level handball [2, 3]. Furthermore, it was pointed out that ball-throw velocity performances in handball are related to upper and lower limb strength and power as well as sprint performance [1, 4]. To this end, various training strategies have been effectively implemented to improve these performance components, including but not limited to strength, plyometric, and elastic bands in young handball players [1, 5, 6].

In recent years, there has been an increasing interest in the use of resisted sprint training among coaches and sports scientists in the training process or research studies for a variety of team sports [7–11]. This training modality involves performing sprint exercises with additional loads such as weight vests, belts, sledges, parachutes, partner towing, uphill running, or on sand. This training aims to increase neural activation and lower limb strength, which in turn enhances sprint velocity without negatively affecting sprint and gestural techniques [12]. Moreover, it has been argued that resisted sprint training allows for more efficient transfer to specific movements [13, 14]. This includes incorporating it into various comprehensive sports training regimens and different sports fitness programs [7–10, 15]. This training modality has been widely studied and applied in various sports, including handball, to improve physical performance and direction abilities in young athletes. Nevertheless, previously published studies are conflicting and inconclusive regarding the effect of resisted sprint training on sprinting, jumping and change of direction performance in different team sports [7, 8, 10]. While some studies have reported positive effects of resisted sprint training on these abilities in different team sports [9–11], others have not found significant improvement [7, 8]. In this context, only a limited number of studies have investigated the effects of resisted sprint training on sprint performance and muscle architecture in female handball players [16]. In addition, there is currently no available information on the effect of this type of training on vertical and horizontal jump performances, change of direction, and ball-throw velocity in handball.

Considering the specificity of handball, it is important to develop strength and explosiveness in both the lower and upper limbs in a coordinated manner, rather than dissociating them. This is particularly crucial given that the activity of the upper limbs, particularly during shooting, is always initiated by a motor action of the lower limbs. Thus, arm-leg coordination should be a primary focus in the physical preparation of handball players. Under the principle of training specificity, as stated by Rumpf et al. [12], it was postulated that the utilization of a weighted belt-resisted sprint training program for the upper and lower limbs would result in a more significant improvement in sprinting, and jumping abilities, change of direction, and ball-throw velocity in pubertal handball players.

To the best of our knowledge, no previous studies have explored the impact of distributed loaded limbs’ resisted sprint training on ball-throw velocity in handball. This particular handball skill is considered a crucial factor in handball performance [1]. It is widely acknowledged that the primary determinants of throwing ball velocity are the timing of movement in consecutive body segments, technique, and the strength and power of both upper and lower limbs [4]. This study aimed to investigate the effect of a resisted sprint-training program using either distributed external load on upper and lower limbs (weighted belt) or vertical (weighted vest) on sprinting and jumping abilities, change of direction, and handball-throw velocity in young handball players over a six-week in-season period, in comparison to un-resisted normal sprint training program. Therefore, we hypothesized that a belt-resisted sprint-training program in young male handball players would result in more significant training-induced adaptation than a vest-resisted sprint or an un-resisted normal sprint-training program.

## MATERIALS AND METHODS

### Experimental Approach to the Problem

**FIG. 1:**
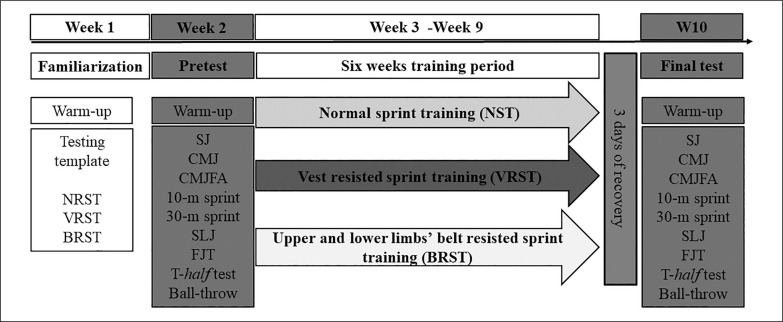
Schematic overview of the study design. SJ: squat jump; CMJ: countermovement jump; CMJFA: free-arm countermovement jump; SLJ: standing long jump; FJT: five jump test.

To explore the substantive research question, we matched adolescent handball players for age, maturation status, field position, height, and training experience. These players were randomly assigned to three training groups: normal sprint training (NST), weighted-vest resisted sprint training (VRST), and upper and lower limbs’ weightedbelt resisted sprint training (BRST). This design has been intended to investigate whether additional vertical or upper and lower extremity loading during sprint training improves skill-related physical performance in young handball players over un-resisted normal sprint training alone. The outcome measures included horizontal and vertical jump (squat jump [SJ], countermovement jump [CMJ], free-arm countermovement jump [CMJFA], standing long jump [SLJ], Five-jump test [FJT]), sprint (10-m and 30-m), change of direction (T-*half* test) and ball-throw velocity performances. The training program lasted 6 weeks and was conducted during the in-season competitive period. During this period, participants train at their club five times per week for 90 minutes. The majority of training sessions focus on technical and tactical skills, accounting for 60–65% of overall training time. Conditioning sessions are conducted twice a week and account for around 35–40% of total training time (Table 1). This program was preceded by 1 week of familiarization and 1 week for the pretest followed by 1 week for the final test (Figure 1). In addition to their standard handball training pattern, all groups performed two sprint sessions with or without additional load. Testing was carried out at the same time of the day, and under the same experimental conditions, at least three days after the last game. Standard verbal encouragement ensured maximal effort throughout was provided for all subjects.

**TABLE 1:** Participant’s characteristics (mean ± SD).

	Age (y)	Body mass (kg)	Height (cm)	% Body fat	Lower limb length (cm)	Upper limb length (cm)	Tanner stage
NST (n = 8)	14.4 ± 0.3	68.1 ± 4.7	173.6 ± 3.0	21.8 ± 0.5	89.3 ± 2.3	70.4 ± 1.5	3.63 ± 0.52
VRST (n = 8)	14.4 ± 0.2	68.6 ± 3.9	177.1 ± 4.1	21.6 ± 0.3	90.6 ± 2.7	70.9 ± 1.9	3.78 ± 0.44
BRST (n = 9)	14.3 ± 0.2	67.7 ± 4.5	176.2 ± 3.0	21.7 ± 0.3	90.3 ± 2.4	70.6 ± 1.3	3.67 ± 0.50
P value (ES)	0.57 (0.34)	0.90 (0.14)	0.12 (0.68)	0.52 (0.37)	0.54 (0.36)	0.82 (0.19)	0.88 (0.16)

NST: normal sprint training; VRST: vest resisted sprint training; BRST: belt resisted sprint training; ES: effect size.

### Participants

Twenty-five young handball players volunteered to participate in this study. All players regularly participated in high-level national competitions. The sample size was calculated a priori according to Beck’s procedures [17] and using the software G*Power [18]. Values for α were set at 0.05 and for power at 96%. Based on the results of Hammami et al. [6] and Aloui et al. [19], effect sizes were estimated to be > 0.8 (Large effect). Eight participants per group would provide maximal chances to minimize the risk of incurring a type II statistical error. The study participants were randomly assigned to either NST (n = 8), VRST (n = 8) or BRST (n = 9) groups. Written consent was obtained from the participant’s parents/legal guardians after being fully informed about the purpose of the study, testing procedures, and potential risks. The study protocol was conducted with the agreement of the Ministries of both Education and Sports (MES-00002022-014) and was performed according to the Declaration of Helsinki (2013), and was approved by the local Institutional Ethical Committee of the University of Sousse.

### Anthropometrics

The anthropometric data of participants are summarized in Table 2. Body mass and height were measured with calibrated devices (Tanita Corporation of America, Arlington Heights, IL, United States, accuracy 100 g, and Harpenden Portable Stadiometer, Pembs, United Kingdom, accuracy 0.1 cm, respectively). Body fat mass (%) was estimated by the four skinfolds method with a clamp mark Harpenden caliper (Harpenden 1124, Groningen, Netherlands) using the revised formula of Durnin and Womersley [20]. In addition, the measurements of each length extremity were performed; participants’ were in a supine position, using a standard tape measure with an accuracy of 0.1 cm. The upper limb length was taken on the dominant arm and was the distance from the tip of the acromion to the wrist flexion crease. The lower limb length was the distance from the anterior superior iliac spine to the medial malleolus. All of the measurements were performed twice by the same evaluator to ensure consistency and the average was retained for analysis. All participants were in puberty and this was determined by pubic hair development according to the Tanner classification [21] at the beginning and the end of the 6-week training period by a trained paediatrician.

**TABLE 2:** Details of weekly training design followed by NST, VRST and BRST groups over the six-week training program.

Day	Objectives
Monday	Recovery session

Tuesday	Conditioning + Technical training + Game Sprint training with and without additional load (NST, VRST, and BRST)

Wednesday	Technical and tactical training + Game

Thursday	Conditioning + Technical training + Game Sprint training with and without additional load (NST, VRST, and BRST)

Friday	Day off

Saturday	Technical and tactical training + Game

Sunday	Match

NST: normal sprint training; VRST: vest resisted sprint training; BRST: belt resisted sprint training.

### 10 and 30-meter sprint test

Sprint performance was assessed at 10-m and 30-m test runs using photocells (HL3-1x Wireless Photocell-TAG-Heuer Professional Timing). The photocells were placed at waist height and the time was measured to one-hundredth of a second. From a standing position 0.3 m behind the starting line, the players initiated the sprint when they were ready and were encouraged to perform each sprint as fast as possible. After one practice trial, each participant had two attempts separated by a 5-minute rest and the best performance was taken for analysis.

### Vertical jump tests

Players had to perform both the squat jump (SJ), the counter-movement jump (CMJ), and the free-arm countermovement jump (CMJFA) according to the standardized protocols described previously [22]. Jump performances were measured using an infrared timing system (Optojump; Microgate SARL, Italy). After one practice trial, each jump was performed twice separated by a 2-minute rest and the best performance was recorded. In the SJ, subjects started with their hands on the hip with a 90° leg-thigh angle. For the CMJ, subjects dropped their body from a standing position by bending their hips and knees during a short pre-stretch counter-movement action, and then immediately extended their hips and knees and jumped as high as possible. For the CMJFA, participants followed the same pattern as the CMJ using their arms in the final phase of the jump. After one practice trial for each test, SJ, CMJ and CMJFA were repeated twice, and the best height in cm was recorded.

### Horizontal jump tests

Each player performed two horizontal jump tests including the 2-footed standing long (SLJ) and the five-jump test (5JT) according to the standardized protocols described previously [22, 23]. For the SLJ test, participants stand behind the starting line with feet together and vigorously push and jump forward as far as possible. The distance is measured from the take-off line to the point where the back of the heel closest to the take-off line lands on the mat or non-slippery ground. For the FJT, each participant began the test with their feet together and had to choose which foot to land first at the beginning of the exercise. Throughout the final stride, the player was asked to finish with their feet together. After one practice trial for each test, SLJ and FJT were repeated twice, and the best result in meters was recorded.

### Change of direction test

Each player performed the T-*half* test according to the standardized protocols described previously [24]. The players initiated to start when they were ready and were encouraged to perform the test as fast as possible. The participant first ran or moved as fast as possible forward to the centre cone, second side-stepped right 2.5 m to the right cone, third side-stepped left 5 m to the far-left cone, and then side-stepped back right to the centre cone. The participant then ran or backed up as fast as possible to cross the finish line. After one practice trial, the T-*half* test was repeated twice, and the best result in seconds was recorded.

### Ball-throw velocity

The ball-throwing velocity was recorded using a radar gun (Sports Radar Gun SRA 3000; Precision Training Instrument, IL) with an accuracy of about 1.24 km/h. According to the protocol previously reported by Laffaye et al. [25], the radar gun was positioned three meters behind the player, in the thrower-target axis at a height corresponding to the player’s height. After a standardized warm-up, participants were given 5 attempts to execute a jump throw using ball standards (circumference 54 cm, mass 325 grams) and a rest of 45-s between throws. Participants performed a jump throw according to the protocol previously described by Aloui et al. [19], starting with a preliminary three-step run before jumping vertically and releasing the ball into the air behind a line 9-m from the goal. Players are instructed to cock their shooting arms and aim for the goal’s centre, with only throws entering the goal without touching the ground are considered valid. The greatest ball-throw velocity was selected for further analysis.

### Training

During the six-week training program, NST, VRST, and BRST performed, in addition to their standard handball training, twice a week additional un-resisted sprint and resisted sprint training using a weighted vest or distributed weighted belt on upper and lower limbs, respectively as recommended by Alcaraz et al. [26] (Table 3). During loading, VRST players wore vests with 12.6% body mass, while RBST players carried 0.22 kg wrist and 1.22 kg ankle belts. Participants were introduced to load-bearing devices the week before training.

**TABLE 3:** Summary of the sprint-training program for NST, VRST and BRST groups. Number of Sets × Number of Sprints in 1 Set × Distance of Each Sprint.

Weeks	Training	Total distance per session	Total distance per week
1–2	3 × 3 × 20-m 2 × 5 × 10-m	280-m	560-m
3–4	3 × 3 × 30-m 2 × 5 × 10-m	370-m	740-m
5–6	3 × 3 × 20-m 2 × 5 × 10-m	280-m	560-m

Rest interval between repetitions and sets is 2 and 5 minutes, respectively.

### Statistical analysis

Data were presented as mean ± SD. Statistical analyses were performed using the SPSS package (SPSS Inc. Chicago. IL. version 20.0). After checking the normality and homogeneity distribution of data using the Shapiro-Wilk and Levene tests, an independent *t*-test was calculated to determine significant differences between groups in baseline values. The variations in the dependent parameters were analyzed by separate mixed-factors ANOVA (time × group) for repeated measurements. Post-hoc analysis using Bonferroni’s correction was then performed to calculate the main effect for group (two levels: Experimental group and Control group) and time (two levels: pre- and post-training). Considering that, in the sample small findings with no statistical significance may present considerable practical relevance; partial eta-squared ($\eta_{\text{p}}^{2}$) was quantified as this is appropriate in the analysis of variance of repeated measures [27]. Effect sizes were classified as small ($\eta_{\text{p}}^{2}$ up to 0.059), medium (between 0.059 and 0.138) and large (greater than 0.138). Additionally, betweengroups standardized mean differences or effect sizes (ES) in pre- and in pre-to-post performance change were calculated using Cohen’s *d* and corrected by Hedge’s g to avoid a biased estimation of the population effect size provided by Cohen’s *d*. According to Cohen, ES can be classified as small (0 ≤ *d* ≤ 0.49), medium (0.50 ≤ *d* ≤ 0.79) and large (*d* ≥ 0.80) [28]. Sprinting, jumping, and ball-throw velocity performances showed an ICC > 0.8 and a CV < 5%. The level of significance was set at p < 0.05.

## RESULTS

All participants in the BRST, VRST, and NST groups underwent the intended training procedure, and none of them reported any injuries related to the training or testing. Before the training, the Shapiro-Wilk and Levene statistics showed anthropometric and physical testing values ranging from 0.93 to 0.98, with p-values ranging from 0.11 to 0.87 for normality and 0.10 to 2.43, with p-values ranging from 0.90 to 0.11 for homogeneity. In addition, there were no statistically significant differences in anthropometric measurements (Table 2) or performance in testing between the three groups (Table 4).

**TABLE 4:** Pre- and post-training program testing performances (mean ± SD).

Variable	Condition	NST (n = 8)	VRST (n = 8)	BRST (n = 9)	$\eta_{\text{p}}^{2}$ (group)/p value	$\eta_{\text{p}}^{2}$ (Time)/p value	$\eta_{\text{p}}^{2}$ (time × group)/p value
SJ height (cm)	Pre	29.7 ± 4.3	26.9 ± 4.1	29.5 ± 4.1	0.10	0.53	0.46
	Post	29.3 ± 4.1	30.0 ± 5.2	34.2 ± 4.8	/	/	/
	Δ (CI 95%)	-0.6 (11.6)^$‡‡^	11.4 (4.5)^**^	16.3 (4.0)^***^	0.31	0.000	0.001
	ES (Cohen’s d)	0.09	0.62	1.0			
	Chances (%)	11/85/4	84/16/0	99/1/0			
	Quantitative assessment	Unclear	Unclear	Very likely			

CMJ height (cm)	Pre	29.5 ± 3.5	28.1 ± 3.8	31.2 ± 3.7	0.23	0.32	0.27
	Post	30.0 ± 3.9	28.9 ± 4.2	34.9 ± 3.7	/	/	/
	Δ (CI 95%)	1.8 (5.6)^‡‡^	3.4 (11.9)^†^	12.0 (3.8)^**^	0.06	0.004	0.03
	ES (Cohen’s d)	0.13	0.18	0.93			
	Chances (%)	5/94/1	16/81/3	99/1/0			
	Quantitative assessment	Unclear	Unclear	Very likely			

CMJFA height (cm)	Pre	34.7 ± 4.2	33.6 ± 3.4	38.3 ± 5.0	0.36	0.37	0.35
	Post	35.2 ± 3.6	34.6 ± 3.9	43.0 ± 4.6	/	/	/
	Δ (CI 95%)	1.7 (6.5)^‡^	3.2 (7.8)^†^	12.9 (7.1)^**^	0.007	0.001	0.009
	ES (Cohen’s d)	0.10	0.26	0.93			
	Chances (%)	6/92/2	21/77/2	99/1/0			
	Quantitative assessment	Unclear	Unclear	Very likely			

SLJ (m)	Pre	2.0 ± 0.2	2.0 ± 0.1	2.1 ± 0.1	0.22	0.67	0.23
	Post	2.1 ± 0.2	2.1 ± 0.1	2.3 ± 0.1	/	/	/
	Δ (CI 95%)	2.7 (4.6)	5.3 (1.7)^***^	6.5 (1.5)^***^	0.06	0.000	0.06
	ES (Cohen’s d)	0.29	1.10	0.97			
	Chances (%)	20/79/1	100/0/0	99/1/0			
	Quantitative assessment	Unclear	Unclear	Very likely			

FJT (m)	Pre	10.1 ± 0.8	10.1 ± 0.4	10.6 ± 0.8	0.24	0.57	0.29
	Post	10.3 ± 0.9	10.6 ± 0.4	11.6 ± 0.9	/	/	/
	Δ (CI 95%)	2.7 (5.5)^‡^	4.3 (2.3)^**^	8.9 (3.6)^***^	0.05	0.000	0.02
	ES (Cohen’s d)	0.28	0.91	1.04			
	Chances (%)	21/78/1	96/4/0	100/0/0			
	Quantitative assessment	Unclear	Unclear	Very likely			

10-m time (s)	Pre	2.06 ± 0.04	2.07 ± 0.08	2.04 ± 0.07	0.13	0.69	0.31
	Post	2.02 ± 0.09	1.98 ± 0.07	1.92 ± 0.08	/	/	/
	Δ (CI 95%)	-1.7 (2.4)^‡^	-4.1 (2.4)^**^	-6.0 (2.1)^**$^	0.22	0.000	0.02
	ES (Cohen’s d)	0.44	1.12	1.5			
	Chances (%)	41/59/0	97/3/0	100/0/0			
	Quantitative assessment	Unclear	Unclear	Very likely			

30-m time (s)	Pre	4.83 ± 0.13	4.81 ± 0.12	4.77 ± 0.20	0.14	0.47	0.51
	Post	4.86 ± 0.21	4.73 ± 0.14	4.61 ± 0.18	/	/	/
	Δ (CI 95%)	0.5 (1.0)^‡‡^	-1.7 (1.1)^*^	-3.5 (0.9)^**^	0.20	0.000	0.000
	ES (Cohen’s d)	0.16	0.57	0.80			
	Chances (%)	11/87/2	66/34/0	98/2/0			
	Quantitative assessment	Unclear	Unclear	Very likely			

T-*half* test (s)	Pre	6.33 ± 0.26	6.30 ± 0.36	6.20 ± 0.31	0.05	0.70	0.02
	Post	6.01 ± 0.41	5.94 ± 0.32	5.80 ± 0.26	/	/	/
	Δ (CI 95%)	-5.0 (4.1)^*^	-5.6 (2.7)^**^	-6.3 (2.3)^***^	0.54	0.000	0.81
	ES (Cohen’s d)	0.87	0.99	1.33			
	Chances (%)	87/13/0	97/3/0	100/0/0			
	Quantitative assessment	Likely	Very likely	Almost certainly			

Ball-throw velocity (km ∙ ^−1^)	Pre	78.6 ± 2.6	78.5 ± 2.2	78.7 ± 2.6	0.13	0.99	0.96
	Post	80.7 ± 2.7	83.6 ± 2.6	88.6 ± 3.0	/	/	/
	Δ (SD)	2.8 (1.0)^$$‡‡^	6.5 (0.5)^***††^	12.5 (3.7)^***^	0.21	0.000	0.000
	ES (Cohen’s d)	0.44	1.12	2.27			
	Chances (%)	36/64/0	100/0/0	100/0/0			
	Quantitative assessment	Unclear	Almost certainly	Almost certainly			

NST: normal sprint training; VRST: vest resisted sprint training; BRST: belt resisted sprint training; Δ: changes (%); ES: effect size; significant difference within group:

* p < 0.5,

** p < 0.01,

*** p < 0.001; significant difference between BRST and VRST:

† p < 0.05,

†† p < 0.01; significant difference between BRST and NST:

‡ P < 0.05,

‡‡ p < 0.01; significant difference between VRST and NS:

$ p < 0.05,

$$ p < 0.01.

The mean values of all tests in pre- and post-training program are illustrated in Table 4. Significant interactions were found (training × group) for SJ, CMJ, CMJFA, SLJ and 5JT performances (F_(2.22)_ = 9.32, $\eta_{\text{p}}^{2}$ = 0.46; F_(2.22)_ = 4.04, $\eta_{\text{p}}^{2}$ = 0.27; F_(2.22)_ = 5.90, $\eta_{\text{p}}^{2}$ = 0.35; F_(2.22)_ = 5.90, $\eta_{\text{p}}^{2}$ = 0.35, respectively). Post-hoc analysis revealed that the performances of SJ, SLJ and FJT increased only in BRST group (p < 0.001) and VRST group (p < 0.01). Moreover, post-hoc analysis revealed that the performances of CMJ and CMJFA increased only in BRST group (p < 0.01). Significant interactions were also found for 10-m and 30-m sprint performances (F_(2.22)_ = 10.88, $\eta_{\text{p}}^{2}$ = 0.25 and F_(2.22)_ = 8.86, $\eta_{\text{p}}^{2}$ = 0.22, respectively). Post-hoc analysis revealed that the performances of the 10-m and 30-m sprints increased only BRST group (p < 0.01) and VRST group (p < 0.01 and < 0.05, for 10-m and 30-m sprints respectively). In addition, a significant interaction was found for handball-throw performance (F_(2.22)_ = 0.96). Post-hoc analysis also revealed that handball-throw performance increased only BRST group (p < 0.001) and VRST group (p < 0.001). No significant interactions were found (training × group) for the T-*half* Test. Post-hoc analysis also revealed that all groups improved change of direction performance with the largest effect (large) observed for the BRST group.

**FIG. 2:**
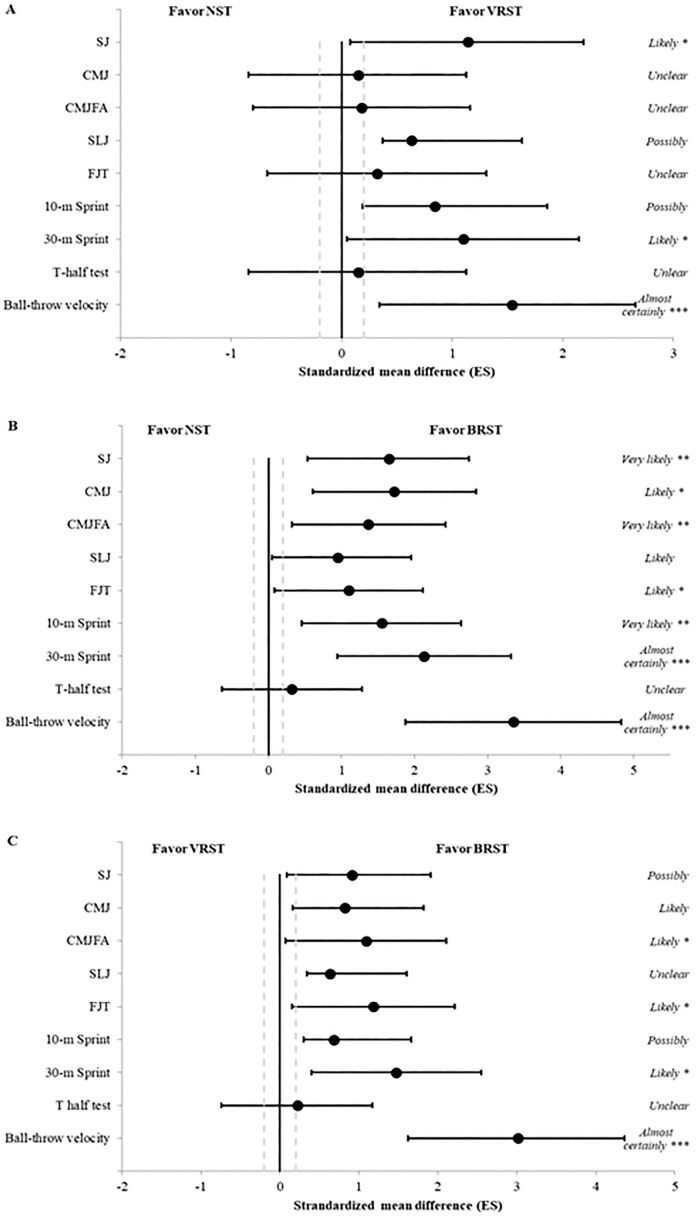
Between-group comparison of sprint, jump, change of direction and ball-throw velocity performance. Bars indicate uncertainly in the true mean changes with 95% confidence intervals. BRST: bled resisted sprint training group; VRST: vest resisted sprint training group; NST: normal sprint training group. SJ: squat jump; CMJ: countermovement jump; CMJFA: free-arm countermovement jump; SLJ: standing long jump; FJT: five jump test; ES: effect size. Inferences are small (0 ≤ *d* ≤ 0.49), medium (0.50 ≤ *d* ≤ 0.79) and large (*d* ≥ 0.80). Qualitative assessment: possibly (25%–75%), *likely (75%–95%), **very likely (95%–99%), and ***almost certainly (> 99%).

Figure 2A, 2B, and 2C showed the between-group comparison on sprinting, jumping ability, change of direction and handball-throw velocity performances. The improvement in 30-m sprint (ES: 1.10 [95% CI: 0.05–2.15]), SJ and ball-throw velocity performances (ES: 1.14 [95% CI: 0.08–2.19] and ES: 1.54 [95% CI: 0.34–2.66]. respectively) were largely greater in VRST group than NST group (Figure 2A). In addition, the improvements in all test performances (ES from 1.10 [95% CI: 0.05–2.12] for FJT to ES: 3.35 [95% CI: 1.88–4.83] for ball-throw velocity) except SLJ, were largely grater in BRST group compared with NST group (Figure 2B). Similarly, between-group comparisons showed that BRST group had the greatest improvement for the 30-m sprint (ES: 1.47 [95% CI: 0.40–2.55]), CMJFA (ES: 1.09 [95% CI: 0.07–2.11]), and ballthrow velocity (ES: 3.01 [95% CI: 1.62–4.40]) performances than VRST group (Figure 2C).

**FIG. 3:**
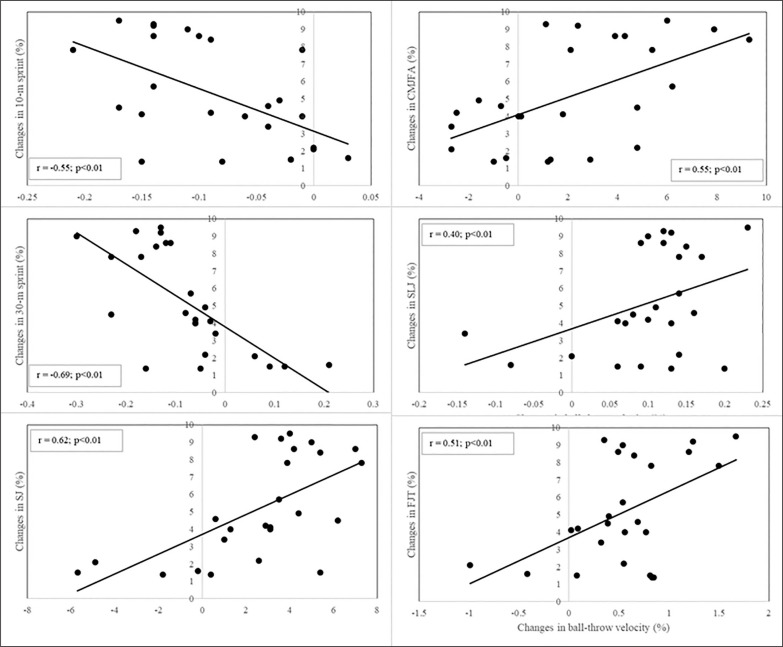
Correlation between changes in SJ, CMJFA, SLJ, FJT and 10-m and 30-m sprint performances and changes in ball-throw velocity. SJ: squat jump; CMJFA: free-arms countermovement jump; SLJ: standing long jump; FJT: five jump test.

When data from all groups were pooled, there were positive relationships between individual percentage changes in ball-throw velocity and individual percentage changes in SJ, CMJFA, SLJ, and FJT performances (r = 0.62, r = 0.56, r = 0.40, and r = 0.51, respectively; p < 0.01). Individual percentage changes in ball throw also showed negative relationships with individual percentage changes in 10-m and 30-m sprint performances (r = -0.55 and r = -0.69, respectively; p < 0.01) (Figure 3).

## DISCUSSION

This study aimed to investigate the effects of a resisted sprint-training program using upper and lower limb distributed loads and weighted vest compared to un-resisted normal sprint training on sprinting, jumping, change of direction abilities and ball-throw velocity in young handball players. The main findings of the present study were the greater effectiveness of upper and lower limbs weighted-belt resisted sprint training compared with weighted vest resisted sprint training or un-resisted normal sprint training for improving sprint and vertical and horizontal jump abilities as well as ball-throw velocity in pubertal handball players.

The results of the sprinting performances showed a significant (group × time) interaction effect for both 10-m and 30-m tests with a substantial effect size observed in the BRST group (ES: 1.55 for 10-m sprint and ES: 2.13 for 30-m sprint). These findings are consistent with previous studies using weighted sledges or weighted vests resisted sprint training in several team sports such as rugby and soccer [10, 11, 29]. Ben Brahim et al. [10] found that six weeks of resisted sprint training with a load of 13% body mass resulted in a 5% increase in 5-m sprint speed in U-19 soccer players after training. In addition, Bachero-Mena and Gonzalez-Badillo [30] assessed the impact of seven weeks of resisted sprint training with weights of 5, 12.5, and 20% body mass, concluding that a load of 20% body mass should be used to enhance the first phase of acceleration up to 30 m. Similarly, Carlos-Vivas et al. [9] found that all resisted sprint training groups, except un-resisted sprint, slightly improved (1.4%–1.9%; ES form -0.43 to -1.0) and 30-m (1.3%–1.5%; ES from -0.37 to -0.76) sprint performances after 8 weeks of training.

The precise mechanisms behind the significant training impact in the BRST group remain unclear. The fundamental principle underlying this training strategy is to develop muscle strength and neural activity through extra load stimulation while maintaining certain movement patterns [31]. In general, it has been suggested that the central nervous system achieves the desirable goal of correcting for increased inertia by modifying muscle activation properties [32]. The BRST group used a load-weighted belt during their resisted training program, possibly increasing the moment of inertia by 50% of their legs [33], resulting in higher contraction velocities in both concentric and eccentric phases of the sprint. This preload stimulus led to postactivation potentiation during sprints with prolonged exposure, leading to greater training responses [15, 29]. Indeed, post-activation potentiation refers to a muscle’s ability to generate force based on previous internal events and the improvement in performance following a submaximal or maximal contraction [34]. Specifically, the induction of these adaptations, along with the potential preservation of the sprinting technique through traditional sprint performance, may have contributed to the speed improvements registered at the BRST group.

Vertical and horizontal jumping are crucial skills in handball, particularly for defensive and offensive tactics like blocking, rebounding, stealing, passing, and shooting [1]. The present vertical and horizontal jump data indicated significant intervention in all groups with a significant (group × time) interaction effect with a significant effect size in players involved in weighted-belt resisted training (ES from 0.96 to 1.72). These results corroborate those of several previous findings [9–11]. Carlos-Vivas et al. [9] compared the effectiveness of horizontal resisted sprint, vertical resisted sprint, combined resisted sprint, and un-resisted sprint training on performance in horizontal and vertical jumps in youth soccer players. They found small to moderate improvement in SLJ performance in players undergoing the different resisted sprint training regimens (1.4% to 4.7%, ES from 0.14 to 0.63). In addition, Chaalali et al. [11] observed significant improvement in SJ, CMJ t (4.23% and 3.59%; ES 0.35 and 0.37, for SJ and CMJ, respectively); and FJT (3.10%; ES = 0.44) performances in young soccer players after six weeks of biweekly partner-towing resisted sprint training. In contrast, the study by Aloui et al. [5] found that junior handball players who underwent 8 weeks of bi-weekly lower limb elastic band-based loaded plyometric training showed significant comparable improvements in SJ (9.4%; ES = 0.83); and CMJ (9.1%; ES = 0.99) performances in experimental and control groups.

The use of upper and lower weighted belts during sprinting in the current study can result in muscular overload, increasing the moment of inertia [33], which in turn probably increases neural recruitment and activation rates during jumps [32]. This leads the nervous system to signal the muscles to contract more efficiently, resulting in greater force during the jump, which is effective in eliciting the meaningful post-activation potentiation effect. The additional resistance of the belt also increases strength in the lower body; specifically the hip extensors, quadriceps, and calf muscles, allowing for greater power generation during the takeoff phase of the jump. These neurophysiological changes, including the increased neural drive to agonist muscles and changes in muscle activation strategies, may enhance the ability to store and release elastic energy during the stretch-shortening cycle [35]. Furthermore, training with additional loads (e.g., a triple extension of the ankle, knee, and hip) during sprint movements with similar biomechanical patterns to jump can improve movement efficiency and movement control for jumping motion [36].

The ability to change direction is a key determinant of handball performance [2]. However, the study found no significant intervention effect (group × time interaction) on change of direction performance, but all groups showed significant time interaction effects, with the weighted vest and belt-resisted sprint training resulting in better improvement. These findings are somewhat expected as all players were involved, in addition to their resisted and un-resisted sprint, into technical and tactical session drills as a part of their normal training program over the present study. Previous studies have shown an improved change of direction ability in response to elastic band training [5, 37] or combined plyometric and short sprint training [6, 19] in young handball players. The lack of response in changeof-direction performance in the present study could be attributed to the limited selection of sprint exercise types or the participants’ similar abilities before the training program. As suggested by Rodríguez-Osorio et al. [38], additional resisted sprint exercises with a change of direction should be included in the training process if the goal is to improve this ability.

The most salient result of the current study is the significant improvement in ball-throwing velocity in all groups after training, with a significant interaction effect (group × time) with a substantial effect size in the weighted-belt resisted sprint training group (ES: 9.55; p < 0.0001). Handball-throwing is a fundamental motor skill involving quick, complex movements and a whole-body kinetic chain from proximal to distal motion [39]. Its accuracy and velocity are crucial for effective scoring [40]. It should be noted that this study is the first to explore the effectiveness of upper and lower limbs weighted belt resisted sprint training in enhancing the throwing performance of pubertal handball players. Several studies applying alternative training strategies for throwing velocity have demonstrated enhanced performance [19, 41, 42]. Mascarin et al. [41] and Bouagina et al. [42] found that strength training with an elastic band and a ballistic training program improved ball throw in young handball players after six and ten weeks of training, respectively. The current study found that resisted sprint training with weight belts significantly improved ballthrow velocity compared to VRST and NST groups. This improvement was significantly associated with enhanced sprinting and jumping abilities after six weeks of bi-weekly resisted sprint training. Indeed, the percentage changes in ball-throw velocity were associated with the changes in the 10-m (r = -55; p < 0.01) and 30-m (r = -0.69; p < 0.01) sprints as well as the horizontal and vertical jumping performance (r = 0.62; r = 0.56; r = 0.40 and r = 0.51, respectively). Our findings support Serrien and Baeyens’ [38] conclusion that handball throwing is a complex movement involving the whole-body kinetic chain from proximal to distal motion. Consequently, the present study’s weighted belt-resisted sprint training program increased the ball-throwing speed of handball players by improving muscular strength and power, motor control, neuromuscular coordination, and structural adaptations in muscles and connective tissues, thus improving their ability to withstand greater forces. Considering the special characteristics of handball, players require agility, quick reactions, and multidirectional movement skills for dynamic, fast-paced games [1–3]. They should also improve shooting accuracy, power under pressure, jumping ability, hand-eye coordination, and spatial awareness to deliver strong shots [1–3]. Training in real handball gameplay situations is a sensible approach to achieve physical fitness improvements suitable for the sport’s demands, as it helps players deliver strong shots over defenders. Nevertheless, the performance gains observed in the current study may be transferred to skill gains even without specific training regimens.

In addition to the explanations provided further up, the study improved sprinting, jumping, and ball-throw performances in both vest and belts-resisted training groups could be explained by the implementation of Alcaraz et al. [26] criteria for resisted sprint training efficiency. Our training program used over 160 m per session and 3700 m for six weeks, with the BRST group showing a greater effect.

The study has some limitations, including not calculating kinetic variables of force and power developed during resisted sprint training, which is crucial for understanding neuromuscular and biomechanical adaptations [14]. Additionally, factors such as knee extensor and shoulder rotator mechanical parameters were not assessed in the study, which could explain muscle adaptation processes elbow extension and shoulder internal rotation torques are primary mechanical contributors to total ball release velocity during standing throws [43]. Future studies ought to investigate how resistance training with weighted belts affects knee extensor isometric parameters and shoulder-related strength of both external and internal rotator torques in young athletes. Furthermore, the inclusion of electromyography activity could provide insights into neuromuscular adaptation.

## CONCLUSIONS

The current study showed that bi-weekly weighted belt-resisted sprint training was more effective than weighted vest-resisted sprint training or un-resisted sprint training for improving sprinting, horizontal and vertical jumping, and ball-throw velocity performances in pubertal male handball players. Handball coaches might incorporate this training form into their gameplay training processes to maximize its benefits and transfer it into specific handball abilities without interfering with their players’ skills.

## Author Contributions

Conceptualization, E.M and M.E; methodology, F.Z, R.H, M.A, and R.T; data collection and statistical analysis, M.A; writing-original draft preparation, E.M., and M.E; writing-review and editing, E.M, F.Z, R.T, and M.E. All authors have read and agreed to the published version of the manuscript.
